# Test–retest reliability of kicking performance assessments over a 2-day interval in elite youth soccer players

**DOI:** 10.7717/peerj.20806

**Published:** 2026-02-17

**Authors:** Jeppe Carstensen, Thomas Bull Andersen

**Affiliations:** 1 Department of Public Health, Section for Sports Science, Aarhus University, Aarhus, Midtjylland, Denmark; 2 AGF A/S, Aarhus, Midtjylland, Denmark

**Keywords:** Youth sports, Reliability, Biomechanics, Soccer, Kicking accuracy, Kicking speed

## Abstract

**Background:**

Reliable assessment of kicking performance is critical for monitoring technical development in youth soccer players. This study examined the reliability of kicking performance assessments in skilled youth soccer players aged 14–18, focusing on maximum ball speed and accuracy.

**Method:**

Using a test-retest design, fifty-three players from a Danish Super League academy completed standardised kicking tests on two occasions separated by 2 days. Maximum ball speed was assessed during penalty-style kicks with a stationary ball, while accuracy was assessed through target-directed kicks with a rolling ball. Two-way mixed-effects intraclass correlation coefficients (ICC) and limits of agreement (LoA) were used to assess reliability.

**Results:**

Results indicated good reliability for maximum ball speed (ICC = 0.87; LoA = [−2.1, 2.5] m/s), consistent with previous studies. Conversely, kicking accuracy demonstrated poor to moderate reliability (ICC = 0.00–0.66; LoA = [−0.46, 0.48] m). Stratified analyses revealed the highest accuracy ICC within the U15 group. Exploratory error ellipse analyses suggested potential utility in assessing kicking distribution, showing good reliability for ellipse angle (ICC = 0.80; LoA = [−0.98, 0.92] rad), though other parameters displayed low reliability. These findings highlight the strengths and limitations of soccer technical assessments. While maximum ball speed tests are highly reliable, kicking accuracy assessments remain problematic and require methodological refinement to improve reliability. Future research should incorporate advanced analytical techniques to enhance test reliability and consistency in elite athlete evaluations.

## Introduction

As the world’s most widely played sport, soccer demands high technical proficiency, with goal-scoring being the decisive factor in match outcomes (Nielsen, 2018). Kicking ability, particularly ball speed and placement accuracy, is crucial for success (Dörge et al., 2002; Katis et al., 2013; Lees & Nolan, 1998). Given the competitive demands of modern soccer, reliable and valid assessments of technical performance are therefore essential for player development and talent identification (Paul & Nassis, 2015).

Assessing soccer performance has been the focus of extensive research. Physical and cognitive performance are measured using well-established, reliable methods (Ali, 2011). While technical assessments of passing, dribbling, and kicking speed demonstrate high reliability and sensitivity (Ali, 2011), kicking accuracy shows limited reliability across studies (Apriantono et al., 2006; Kellis, Katis & Vrabas, 2006; Torreblanca-Martínez et al., 2020; Ferraz, Van Den Tillaar & Marques, 2012; Ferraz et al., 2019; Russell, Benton & Kingsley, 2010; Markovic, Dizdar & Jaric, 2006; Carstensen et al., 2024).

Reliability, defined as the consistency of repeated measurements under identical conditions, is fundamental for longitudinal monitoring in youth soccer (Ali, 2011; Barrow & McGee, 1979). High reliability is essential for distinguishing true changes in skill from measurement error and is therefore a key prerequisite for player monitoring and talent development. However, several existing kicking accuracy tests exhibit moderate to low reliability (*e.g.*, ICC values below 0.5) (Rodriguez-Giustiniani, Rollo & Galloway, 2022; Ali et al., 2007), likely due to the complexity of the tests and insensitive evaluation methods.

Common accuracy tests that divide the goal into scoring zones offer only coarse spatial resolution and are affected by players’ risk-taking behaviour; low scores may reflect conservative shot selection rather than poor accuracy (Torreblanca-Martínez et al., 2020; Stone & Oliver, 2009; Engler, Hohmann & Siener, 2023; Islam, Kundu & Saha, 2019; Gardasevic & Bjelica, 2019; Alkhawaldeh, 2022; Radman et al., 2016; Smith et al., 2016; Dambroz, Clemente & Teoldo, 2022). Alternative methods, such as calculating distance to a target, are more sensitive but often require resource-intensive video analysis, which restricts their feasibility in applied settings (Ferraz et al., 2019; Russell, Benton & Kingsley, 2010; Radman et al., 2016; Finnoff, Newcomer & Laskowski, 2002; Bjelica, Popovic & Petkovic, 2013; Bacvarevic et al., 2012). Conversely, assessments emphasising ecological validity, such as the Loughborough Soccer Shooting Test (LSST), replicate game-like scenarios (Ferraz et al., 2019; Ali et al., 2007; Finnoff, Newcomer & Laskowski, 2002). However, the very features that increase ecological validity (*e.g.*, variable shooting positions, decision-making and time pressure) introduce substantial situational noise, reducing reliability and making it difficult to isolate the specific contribution of shooting technique to performance (Ali, 2011; Russell, Benton & Kingsley, 2010; Ali et al., 2007). As a result, highly ecological shooting tests may demonstrate good face validity but yield unstable measures of accuracy because scores are strongly influenced by momentary tactical decisions and contextual constraints rather than underlying kicking ability. Taken together, these limitations indicate a methodological gap between overly simple and overly complex tests. Intermediate protocols that preserve basic game constraints while controlling key sources of situational variability, such as advancing the ball before striking (Radman et al., 2016), may provide a more appropriate balance between realism, reliability and practicality.

To further overcome these limitations, recent work has applied error ellipse analysis to kicking performance. This approach models the bivariate distribution of shots, quantifying accuracy and variability in both horizontal and vertical dimensions through ellipse parameters such as area, aspect ratio, and orientation (Hunter et al., 2018). These measures provide a richer description of shot distribution and have shown promise in capturing effects of factors such as leg dominance and fatigue that are not evident through traditional accuracy measures (Carstensen et al., 2024; Bjelica, Popovic & Petkovic, 2013; Shimotashiro & Shinya, 2024).

Despite such contributions, the reliability of accuracy assessments in youth players remains underexplored, as most studies have focused on adults (Ali, 2011; Markovic, Dizdar & Jaric, 2006; Radman et al., 2016). Limited evidence in adolescent cohorts highlights methodological concerns. Vieira et al. (2018) assessed accuracy and velocity simultaneously in players from U9 to U20, but their simultaneous evaluation of these attributes confounded interpretation, as run-up speed and constraints on ball velocity influence accuracy (Andersen & Dörge, 2011). Bacvarevic et al. (2012) tested accuracy in 12–13-year-olds without velocity constraints, limiting ecological validity, since slow but accurate shots may not reflect match demands.

Systematic evaluations are particularly scarce in the later developmental stages (14–18 years), which are critical for talent identification and selection (Markovic, Dizdar & Jaric, 2006; Bacvarevic et al., 2012). Reliability in this age group is typically lower than in adults, due to greater variability in technical execution and ongoing motor development (Paul & Nassis, 2015; Buchheit & Mendez-Villanueva, 2013). This highlights the need for robust and standardised test methods. However, Buchheit & Mendez-Villanueva (2013) found that reliability improves as participants surpass peak height velocity (PHV). Accordingly, the present study focuses on academy players aged 14–18 (U15, U17, U19), where most players are post-PHV.

While error ellipse analysis has demonstrated potential for capturing aspects of kicking performance relevant to skill monitoring and talent development (Carstensen et al., 2024; Bjelica, Popovic & Petkovic, 2013; Shimotashiro & Shinya, 2024), the reliability of ellipse-based metrics in elite adolescent players has not yet been established. To our knowledge, no prior study has quantified the test–retest reliability of ellipse-based accuracy metrics in youth academy players.

To address this gap, the study evaluates the reliability of error ellipse metrics and maximum ball speed in academy-level adolescent players, aiming to provide robust methods for technical performance assessment.

## Materials and Methods

### Study design

The study was conducted with a test-retest design in which participants performed the same kicking test separated by two days. Participants were experienced soccer players performing a familiar task—kicking a ball ten times for accuracy in their usual boots on a standard surface. Therefore, we did not anticipate meaningful learning or order effects and did not include a separate familiarisation session before Day 1.

### Participants

Fifty-three skilled youth soccer players were recruited from a Danish Super League football club academy. All players competed at elite national youth level, representing the highest tier of national youth competition. To minimise fatigue-related effects, all testing sessions were scheduled at least 48 h after the players’ most recent match, and no training sessions were undertaken earlier on the day of testing.

The study was conducted in accordance with ethical research guidelines and participation was voluntary. Information about the study was communicated and written parental consent obtained through the football club. Ethical approval for the methods used for data collection and storage was obtained from the Aarhus University faculty research ethical committee (Forskningsetisk Underkomité Health), journal number 2025-0799558, reference number 2025-005

Descriptive characteristics of the participants are presented in Table 1.

**Table 1:** Descriptive characteristics of the participants. Data is reported as mean (SD).

	N	Height (cm)	Weight (kg)	Age (years)
**Participants**	53	180.23 (7.7)	67.99 (10.62)	15.58 (1.52)

### Kicking performance

Kicking performance was assessed exclusively for the dominant leg as the non-dominant leg is considered to be of a lower skill level and to exhibit greater variability in performance (Katis et al., 2013; Bjelica, Popovic & Petkovic, 2013; Shimotashiro & Shinya, 2024).

Testing took place in an indoor biomechanics laboratory equipped with an artificial grass surface (GreenFields MX NF v.2.0, Netherlands), using a Select Brilliant Super ball inflated to 0.9 bar. Participants wore their regular soccer boots and were instructed to use the same pair on both test days, in line with findings from Sterzing and Hennig (Sterzing & Hennig, 2008). Prior to testing, participants completed a standardised warm-up protocol consisting of 5 min of cycling at a self-selected intensity, 20 leg swings with the kicking leg (10 in the sagittal plane and 10 in the transverse plane), and three warm-up kicks performed at a self-selected intensity.

### Maximum ball speed

Maximum ball speed was recorded using a Supido Sports Speed Radar during three 11-metre kicks with a stationary ball. The speed radar was positioned directly behind a suspended net containing the target. Participants were instructed to execute instep kicks with maximal power, with a 30-second rest interval between kicks. The highest ball speed achieved was used for the analysis of all kicking performance variables. If a participant demonstrated progressive improvement across the three attempts, we asked them to perform additional kicks until no further improvement was observed, as this procedure has previously yielded sensitive and reliable measurements of maximal ball speed (Carstensen et al., 2024). The participants performed a maximum of five kicks.

### Accuracy

Kicking accuracy was assessed independently of maximum ball speed, in accordance with findings by Andersen & Dörge (2011). The procedure was conducted similarly to Carstensen et al. (2024). Accuracy was measured using a two-camera system to determine the distance between the ball impact location and the target, represented by a cross suspended at a height of 1.60 metres. Participants performed a total of 10 kicks from a distance of 11 metres, with 30-second rest intervals between attempts (Radman et al., 2016). Consistent with the protocol described by Radman et al. (2016), the ball was rolled forward and kicked while in motion. Based on the findings of previous studies (Hunter et al., 2018; Andersen & Dörge, 2011), participants were instructed to kick at no less than 75% of their measured maximum ball speed. Kicks that did not meet this speed threshold were excluded from the accuracy analysis, and the participants repeated those attempts. Ball speed was recorded using the same radar system as in the maximum ball speed test.

**Figure 1:**
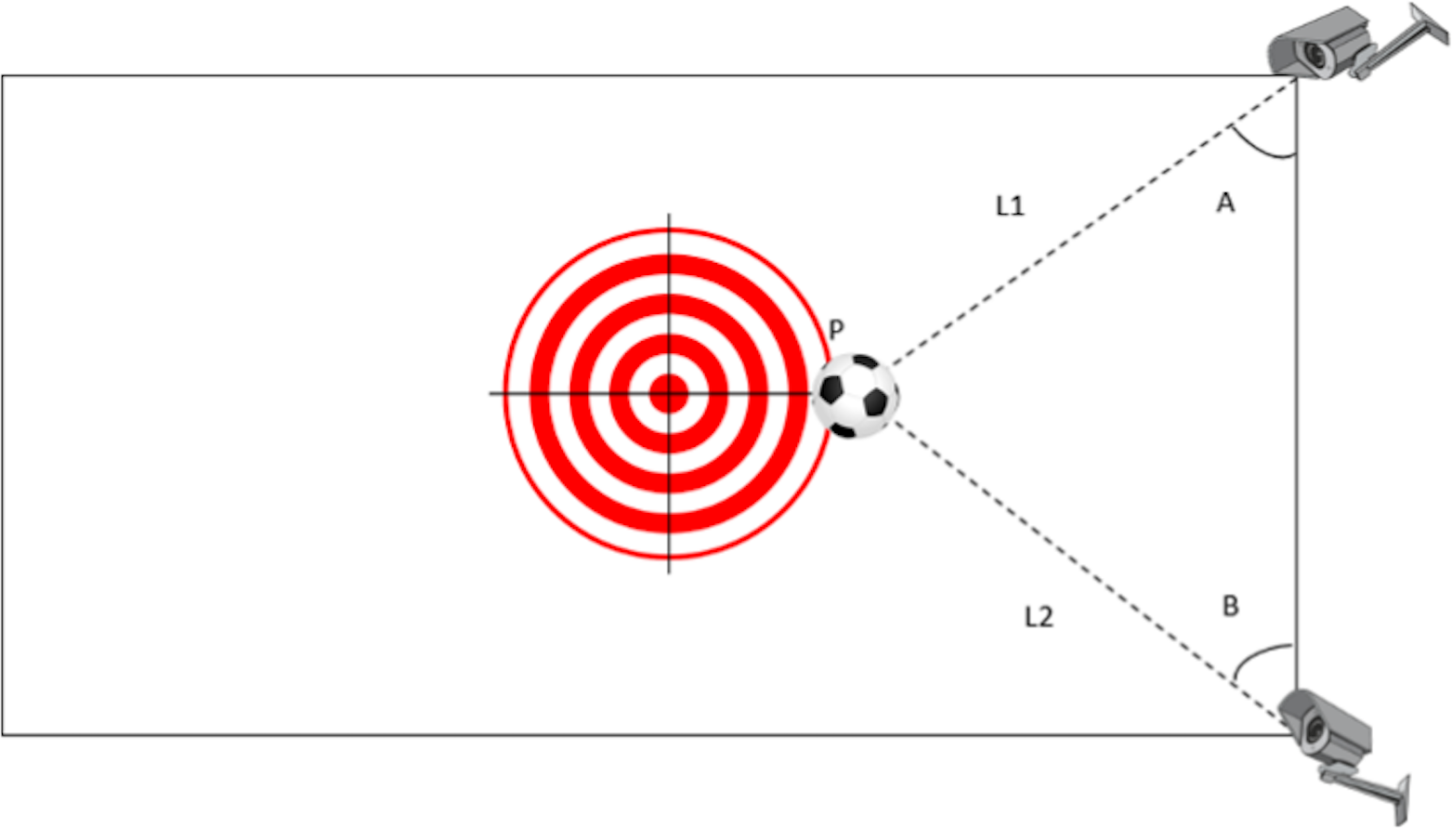
Illustration of accuracy measuring setup. Illustration of the accuracy measurement setup. Two cameras (C1 and C2) record the ball as it passes the target at point P, with the centre of the target representing the intended aiming point. Based on the pixel coordinates from each camera, angles A and B are calculated and used to derive the segment lengths L1 and L2 by triangulation, thereby determining the exact position of P. Kicking accuracy is then quantified as the distance between the target centre and point P. Source credit: Carstensen et al. (2024) CC BY 4.0.

The two-camera system comprised two wall-mounted cameras (Brio 4K Ultra HD Webcam, Logitech) positioned to capture the target cross plane perpendicular to the kicking direction (Fig. 1). The point of ball impact was determined *via* triangulation of pixel coordinates from the two cameras and subsequently analysed using MATLAB R2021a (The MathWorks, Natick, MA, USA). The system was calibrated to an accuracy of 0.016 m.

To further examine kicking accuracy, kick dispersion was analysed using bivariate distributions following the methodology of Hunter et al., (2018). For each kick, the impact location was recorded as Cartesian coordinates (x, y) in metres, and a 95% error ellipse was computed for each participant and test day separately. For the analysis, data from left-footed kickers were mirrored and combined with those from right-footed kickers.

### Statistics and data analysis

Systematic error was assessed using paired *t*-tests with a significance level of 5% to compare results between the two test days. Differences in ball speed and kicking accuracy between age groups were examined using independent two-sample *t*-tests. We inspected quantile–quantile (Q–Q) plots to verify the assumption of normality.

Reliability and random error were assessed using a two-way mixed-effects intraclass correlation coefficient (ICC(2,1)) for absolute agreement, following the guidelines of Koo & Li (2016). To compliment the ICC an additional within-subject coefficient of variation (CV%) analysis was conducted.

Limits of agreement and heteroscedasticity were assessed visually using Bland-Altman plots. Given the sample size (>100 degrees of freedom), we considered bias within the limits of agreement negligible (Hopkins, 2000). All statistical analysis were performed in MATLAB R2021a (MathWorks, USA).

## Results

All 53 participants completed both trial days. Kicking results from each trial day, as well as between-day analyses are presented in Table 2.

**Table 2:** Kicking performance across the two test days. Values are presented as mean (SD) for each day and as mean difference (95% CI) between days. Test-retest reliability was quantified using intraclass correlation coefficients (ICC), and systematic between-day differences were examined with paired *t*-tests. Accuracy is expressed as the average absolute distance (m) between the ball impact location and the centre of the target across all kicks.

	Ball speed (m/s)	Accuracy (m)	Area (m^2^)	Angle (rad)	Long axis (m)	Short axis (m)
Day 1	29.2 (2.30)	0.93 (0.21)	0.40 (0.34)	1.15 (0.75)	0.80 (0.43)	0.15 (0.075)
Day 2	29.01 (2.26)	0.92 (0.20)	0.39 (0.30)	1.18 (0.78)	0.77 (0.38)	0.155 (0.087)
ICC (95% CI)	0.87 [0.78; 0.92]	0.31 [0.05; 0.54]	0.20 [−0.07; 0.45]	0.80 [0.68; 0.88]	0.035 [−0.24; 0.30]	0.31 [0.04; 0.53]
Absolute difference (95% CI)	0.21 [−0.12; 0.53]	0.013 [−0.05; 0.08]	0.01 [−0.10; 0.11]	−0.03 [−0.16; 0.10]	0.03 [−0.13; 0.18]	−0.01 [−0.035; 0.019]
*P*-value	0.20	0.67	0.87	0.66	0.71	0.52
Diff [95% LOA]	0.21 [−2.10; 2.52]	0.013 [−0.46; 0.48]	0.01 [−0.78; 0.80]	−0.03 [−0.98; 0.92]	0.03 [−1.07; 1.13]	−0.01 [−0.20; 0.18]
CV%	2.86	18.37	72.43	29.36	50.70	44.96

Between-day analyses of the 53 participants stratified in the 3 teams are presented in Table 3. No differences between days within the groups were observed.

**Table 3:** Team kicking performance across the three age-group squads. Data are presented as mean (95% CI) for ball speed (m/s) and accuracy (m), where accuracy represents the average absolute distance between the ball impact location and the centre of the target.

	N	Ball speed ICC (95% CI)	Speed difference (95% LOA)	Ball speed CV%	Accuracy ICC (95% CI)	Accuracy difference (95% LOA)	Accuracy CV%
U15	16	0.76 [0.43; 0.92]	0.06 [−2.66; 2.69]	6.84	0.66 [0.24; 0.87]	0.06 [−0.31; 0.43]	21.65
U17	15	0.87 [0.65; 0.96]	0.37 [−2.01; 2.76]	8.02	0.057 [−0.47; 0.55]	−0.03 [−0.50; 0.44]	15.85
U19	22	0.44 [0.02; 0.73]	0.24 [−1.79; 2.26]	2.71	−0.15 [−0.53; 0.29]	0.01 [−0.53; 0.55]	12.37

**Figure 2:**
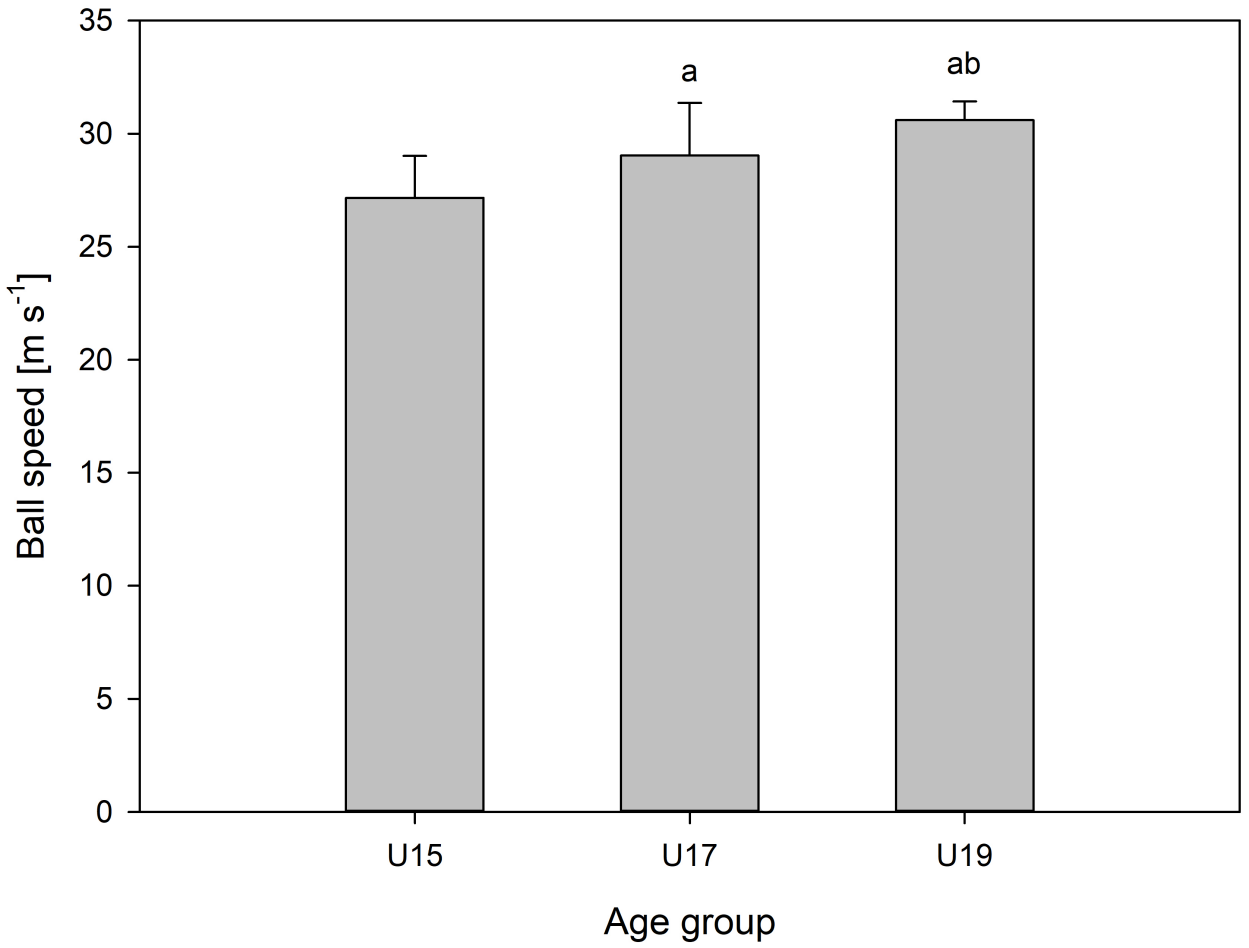
Maximal kicking speed of the three teams. Maximal ball speed (mean ± SD, m/s) for the three age-group squads: U15 (*n* = 16), U17 (*n* = 15), and U19 (*n* = 22). Letters denote significant between-team differences (*p* < 0.05): a indicates a significant difference from U15, and b indicates a significant difference from U17.

Maximum ball speed of the three teams involved is illustrated in Fig. 2. All teams significantly differed from one another. Maximum ball speed was greater for the U19 team than in both U17 and U15, with differences of 1.56 [0.47; 2.65] m/s and 3.44 [2.53; 4.34] m/s respectively.

Maximum ball speed for the U15 team was lower than the U17 and U19, with differences between U15 and U17 of 1.76 [0.24; 3.51] m/s.

**Figure 3:**
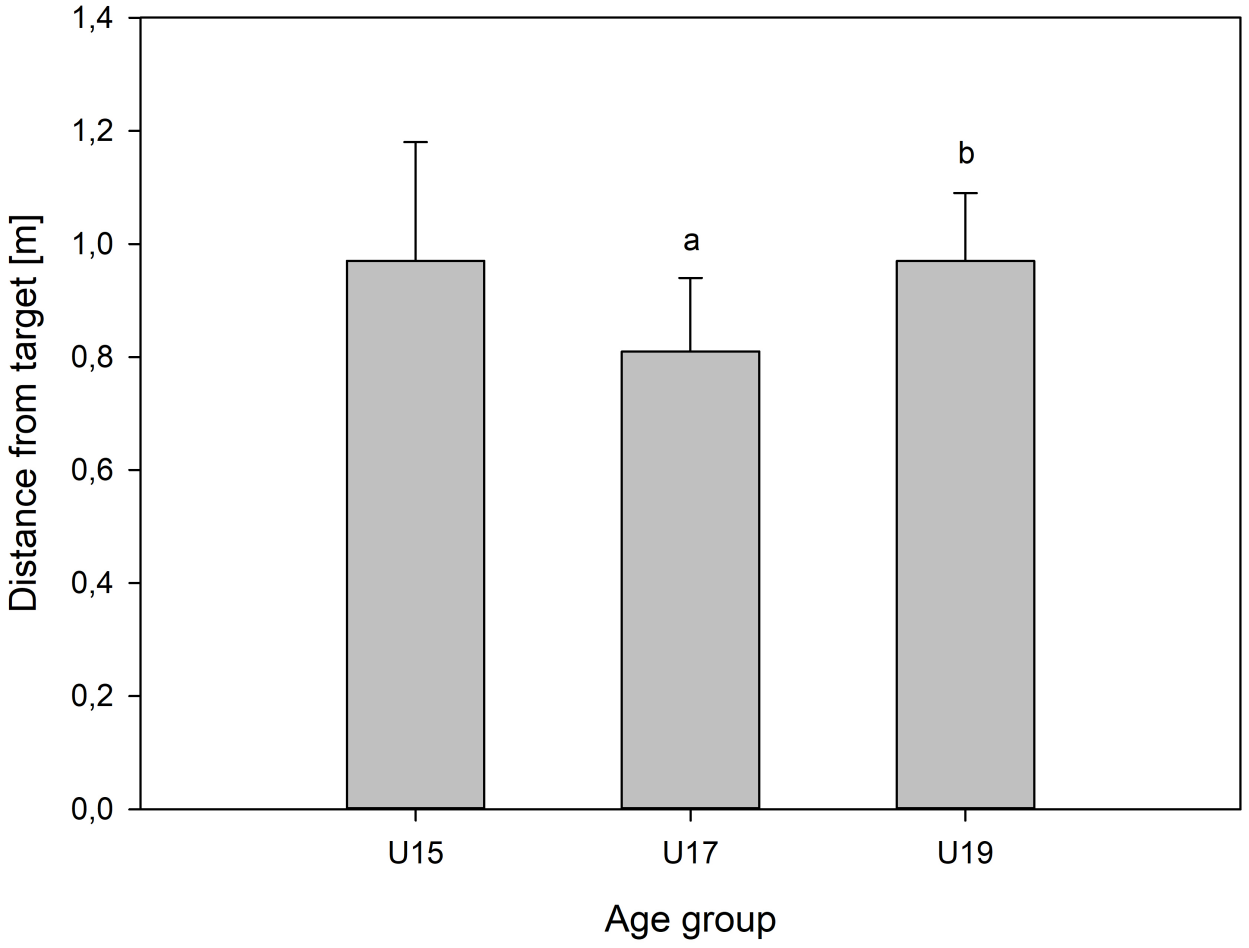
Team kicking accuracy. Kicking accuracy (mean ±SD, m) for the three age-group squads expressed as the average absolute distance between the ball impact location and the centre of the target for U15 (*n* = 16), U17 (*n* = 15), and U19 (*n* = 22). Letters denote significant between-team differences (*p* < 0.05): a indicates a significant difference from U15, and b indicates a significant difference from U17.

Shooting accuracy for the 3 teams is presented in Fig. 3. Between-team comparisons showed that the U17 team was significantly more accurate that the other teams, with mean differences of −0.16 [−0.29; −0.03] m and −0.16 [−0.25; −0.07] m for the U15 and U19 teams, respectively.

No difference between the U15 and the U19 teams was observed, with a mean difference of 0.0 [−0.12; 0.12] m.

## Discussion

The present study evaluated the reliability of kicking assessments in youth soccer players and explored the use of recently introduced distribution metrics.

The results indicated moderate-to-good reliability for maximum ball speed (ICC = 0.87 [0.78, 0.92]). This is consistent with previous findings in young and adult populations using similar test setups, reporting intra-class correlations between 0.70 and 0.94 (Ferraz et al., 2019; Markovic, Dizdar & Jaric, 2006; Radman et al., 2016; Bacvarevic et al., 2012). In contrast, the reliability of kicking accuracy ranged from poor to moderate (ICC = 0–0.66) (Koo & Li, 2016). This reliability is lower than reported ICCs >0.80 in adult samples employing simpler setups (Ferraz et al., 2019; Radman et al., 2016), but comparable to more complex methods, which report ICCs of 0.26–0.38 (Russell, Benton & Kingsley, 2010; Ali et al., 2007). To aid interpretation, ICC values between 0.60 and 0.75 were considered to indicate moderate reliability that may be acceptable for group-level research, whereas values ≥0.75 were interpreted as good reliability likely suitable for most field applications. These thresholds are in line with commonly used classifications of ICC magnitude (Koo & Li, 2016). Complementing these ICC findings, coefficients of variation indicated low relative variability for maximum ball speed (CV = 2.86%) but substantially higher variability for accuracy (CV = 18.37%). The CV measures followed the same trend, whereby increased test complexity was associated with increased variability, consistent with the literature (Rodriguez-Giustiniani, Rollo & Galloway, 2022; Ali et al., 2007; Radman et al., 2016).

Beyond these conventional measures, this study also examined the reliability of error ellipse parameters, representing a novel approach to describing the distribution of kicks introduced by Hunter et al. (2018). In this analysis, the parameters of area (ICC = 0.20 [−0.07; 0.45]), long axis (ICC = 0.035 [−0.24; 0.30]), and short axis (ICC = 0.31 [0.04; 0.53]) all showed poor reliability. The coefficients of variation reinforced this pattern, with very high values for area (CV = 72.43%), long axis (CV = 50.70%) and short axis (CV = 44.96%), indicating substantial trial-to-trial variability in the extent of the distribution.

However, the angle (indicating the tilt of the distribution) demonstrated good reliability (ICC = 0.80 [0.68; 0.88]).

The reliability of this parameter may be explained by factors influencing the tilt of the error ellipse. We propose that the kicking distribution angle reflects foot orientation at ball contact. When players use a consistent kicking technique, this angle may show limited variation because lower-extremity anthropometric characteristics (*e.g.*, foot length, typical range of hip rotation) are relatively constant. Moreover, existing evidence indicates that foot orientation at ball impact is systematically associated with ball flight direction and differs across shooting techniques (Inoue, 2024; Kimachi et al., 2024).

This stability distinguishes the ellipse angle from other parameters (area, long axis, short axis), which appear more sensitive to trial-by-trial inconsistencies in execution.

For researchers, the kicking distribution angle could therefore serve as a proxy measure of technical execution, allowing for a clearer distinction between errors caused by mechanical inconsistencies and those stemming from tactical decision-making or situational constraints. For practitioners, the ellipse angle offers a potential biomechanical marker of directional control in kicking performance, which could be applied in talent development and training interventions. However, the extent to which the orientation of the error ellipse directly reflects the orientation of the kicking foot at impact remains uncertain and requires dedicated empirical testing. In contrast, ellipse area and axis lengths may be more susceptible to day-to-day variability, limiting their reliability for longitudinal monitoring.

Taken together, these findings suggest that among ellipse-derived measures, the angle parameter shows the greatest promise as a tool for assessing directional consistency in youth soccer kicking. However, further validation is required to confirm its sensitivity to factors such as training adaptations, fatigue, or pressure conditions.

Several factors may explain the relatively modest reliability of accuracy measures. First, reliability in this age group (14–18 years) is typically lower than in adults due to ongoing motor development, as outlined in the introduction (Paul & Nassis, 2015; Buchheit & Mendez-Villanueva, 2013).

Second, differences in test sensitivity are important. For example, Radman et al. (2016) reported high reliability (ICC = 0.84) using a design where players advanced the ball before striking. However, the scoring system used large goal boxes (48.8 × 48.8 cm), limiting spatial resolution. If shot variation was smaller than the scoring resolution, between-day differences would remain undetected, artificially inflating reliability. A similar effect may explain the high ICCs reported when only the best attempts were included (Radman et al., 2016) or when unsuccessful kicks were excluded (Bacvarevic et al., 2012). By reducing intra-individual variability, such approaches inflate reliability estimates but at the cost of sensitivity. In contrast, the present study applied stricter criteria and finer measurement resolution (accuracy of 0.016 m), which likely contributed to lower ICCs but offer a more realistic representation of performance consistency and test sensitivity. This interpretation is supported by the limits of agreement (LoA), which indicated relatively narrow ranges for both accuracy [−0.46; 0.48 m] and ball speed [−2.1; 2.5 m/s], comparable to or smaller than those reported in prior work (Russell, Benton & Kingsley, 2010; Markovic, Dizdar & Jaric, 2006; Ali et al., 2007).

Third, sample homogeneity likely constrained ICC values, as they are sensitive to between-subject variance (Hopkins, 2000). In our cohort, no significant differences in accuracy were found between U15 and U19 players, whereas U17 players differed from both. When all teams were pooled for the main analysis, the limited between-player variability reduced ICCs for accuracy. In contrast, maximum ball speed differed significantly between all three age groups and showed smaller relative measurement error (CV error 0.6–2.1%), which contributed to generally higher ICCs for this parameter.

Together, these findings indicate that the observed differences in ICCs between age groups are driven primarily by differences in between-player variability, rather than by poorer measurement precision, and illustrate how restricted variance in relatively homogeneous elite squads can reduce ICC estimates. While broader sampling across skill levels could increase ICC estimates, this would come at the cost of ecological relevance for elite player assessments. This analysis therefore prioritised maintaining a representative academy-level cohort. However, given the relatively small sample sizes within each age group, conclusions from the subgroup analyses should be interpreted cautiously, as they are more susceptible to random error and bias. The subgroup analyses should therefore be viewed as exploratory and primarily as support for the interpretation that differences in ICCs are largely attributable to between-player variability rather than measurement error.

The present study highlights the importance of balancing test complexity with ecological validity in youth soccer kicking assessments. Simpler tests, such as stationary ball kicks, yield higher reliability but have limited game relevance, whereas more dynamic or complex protocols introduce variability that can reduce reliability. Limits of agreement analyses indicated consistent ball speed but persistent challenges in assessing accuracy, reinforcing the need for refined protocols that balance reliability, ecological validity, and practical applicability. Error ellipse analysis offers a novel tool to characterise kicking distribution, and the angle, which demonstrated good reliability, may represent a useful metric of directional accuracy. Additionally, the ellipse captures the spatial distribution and predominant direction of error beyond simple mean distance, providing information on directional control. This knowledge can directly inform aiming strategies in applied contexts (*e.g.*, passes into constrained spaces or penalty kicks). Future research should aim to refine these protocols, incorporating advancements such as error ellipse analysis to enhance both reliability and practical applicability in elite youth soccer contexts.

## Conclusion

This study evaluated the reliability of a youth soccer kicking assessment incorporating newly introduced distribution metrics. Maximum ball speed demonstrated moderate to good reliability, consistent with previous research, whereas accuracy showed poor to moderate reliability.

The error ellipse analysis provided a novel approach to describing kicking distribution. While most ellipse parameters exhibited poor reliability, the angle demonstrated good reliability (ICC = 0.80), suggesting that it may warrant further investigation as a metric for assessing accuracy and distribution patterns, although further validation is required.

The limits of agreement analysis indicated consistent ball speed but highlighted persistent challenges in assessing accuracy. The findings highlight the trade-offs between test simplicity, ecological validity, and measurement precision. Future research should aim to refine assessment protocols and metrics to optimise reliability while maintaining relevance for youth talent development and evaluation in soccer.

## Supplemental Information

10.7717/peerj.20806/supp-1 Supplemental Information 1 Bland altman plot of kicking accuracy

10.7717/peerj.20806/supp-2 Supplemental Information 2 Bland altman plot of error ellipse area

10.7717/peerj.20806/supp-3 Supplemental Information 3 Bland altman plot of maximum ball speed

10.7717/peerj.20806/supp-4 Supplemental Information 4 Bland altman plot of error ellipse angle

10.7717/peerj.20806/supp-5 Supplemental Information 5 Bland altman plot of error ellipse short axis

10.7717/peerj.20806/supp-6 Supplemental Information 6 Bland altman plot of error ellipse long axis

## Additional Information and Declarations

### Data Availability

The following information was supplied regarding data availability: The data is available at Zenodo: Carstensen, J., & Andersen, T. B. (2025). Reliability of Kicking Speed and Accuracy in Youth Soccer Players (Raw Data and Analysis Code) [Data set]. Zenodo. https://doi.org/10.5281/zenodo.16927375.
