# Supplementary figures and images for: Test–retest reliability of kicking performance assessments over a 2-day interval in elite youth soccer players
Source: PeerJ. 2026 Feb 17;14:e20806. doi: 10.7717/peerj.20806 (PMC12922589; doi:10.7717/peerj.20806)

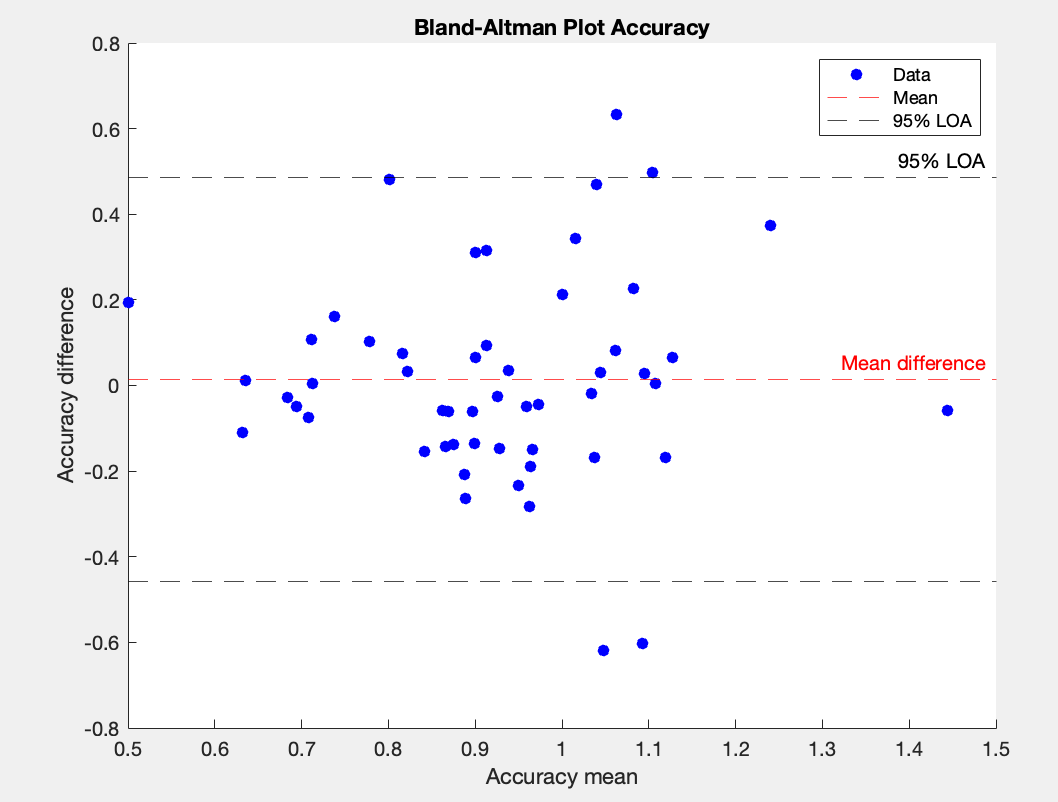

Supplement: Supplemental Information 1 [file peerj-14-20806-s001.png]

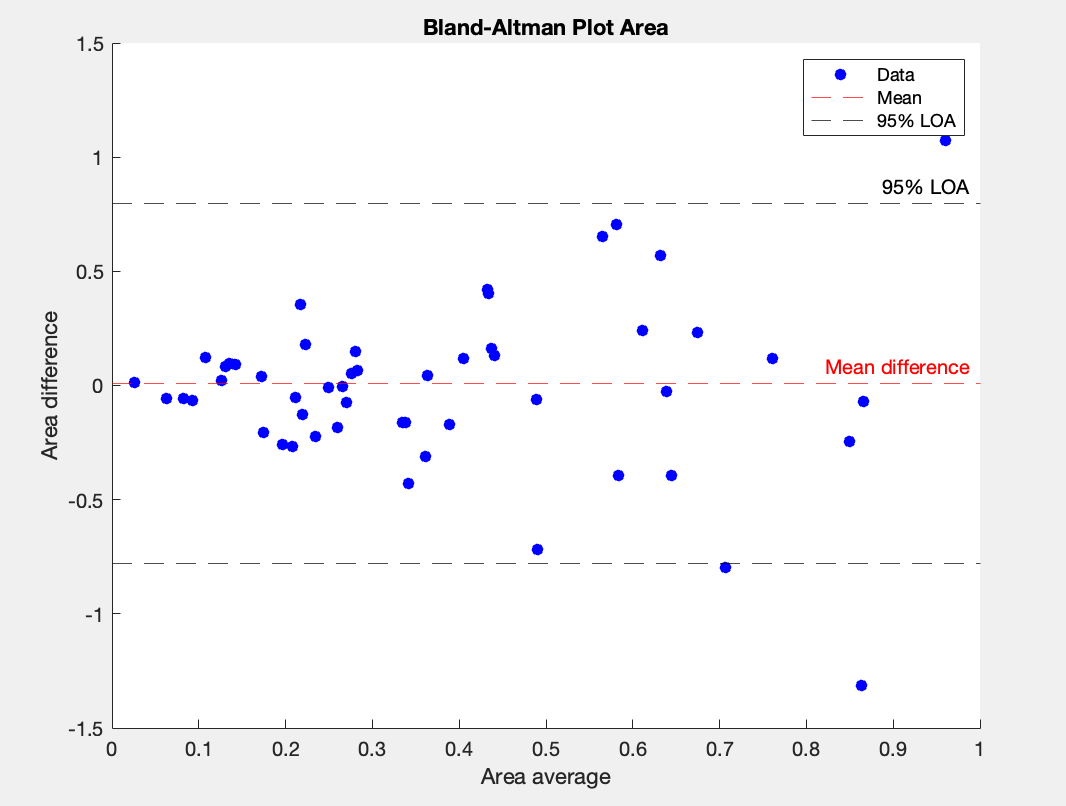

Supplement: Supplemental Information 2 [file peerj-14-20806-s002.png]

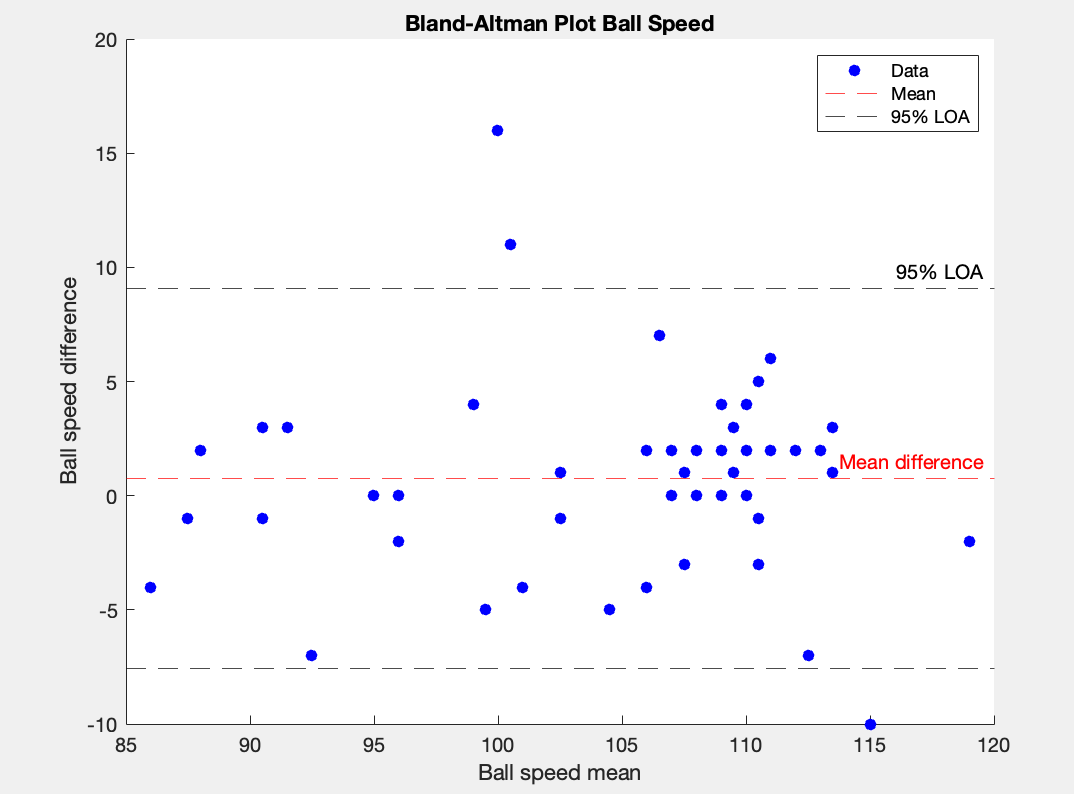

Supplement: Supplemental Information 3 [file peerj-14-20806-s003.png]

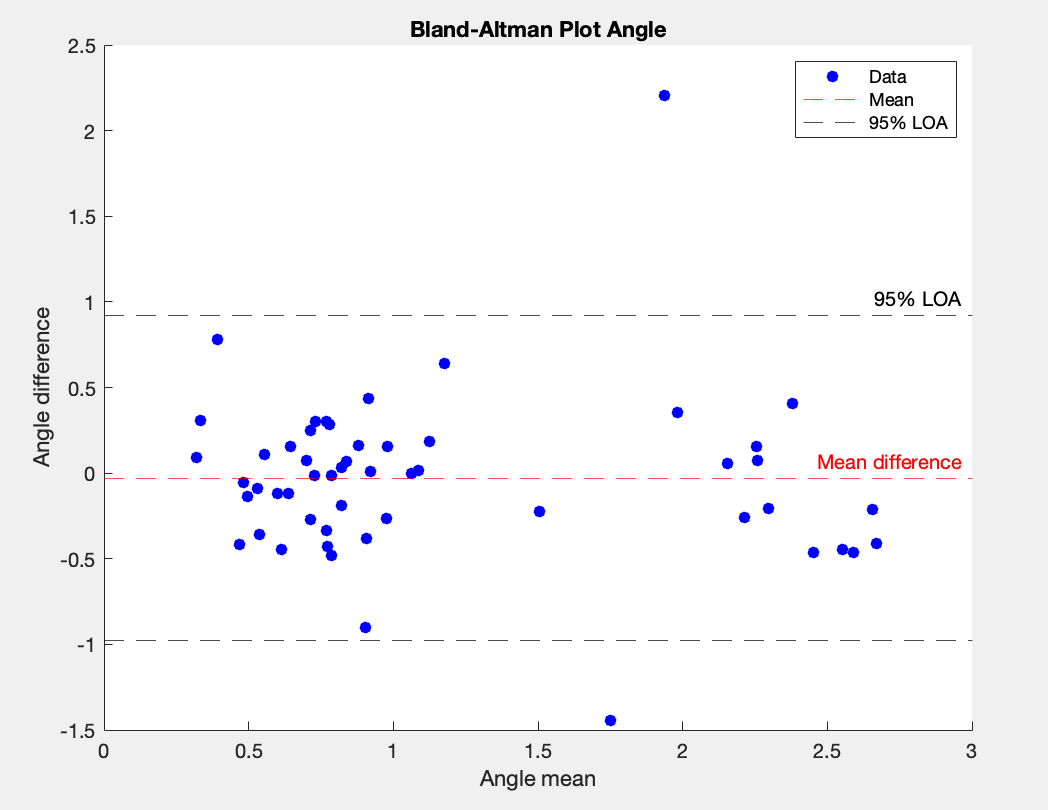

Supplement: Supplemental Information 4 [file peerj-14-20806-s004.png]

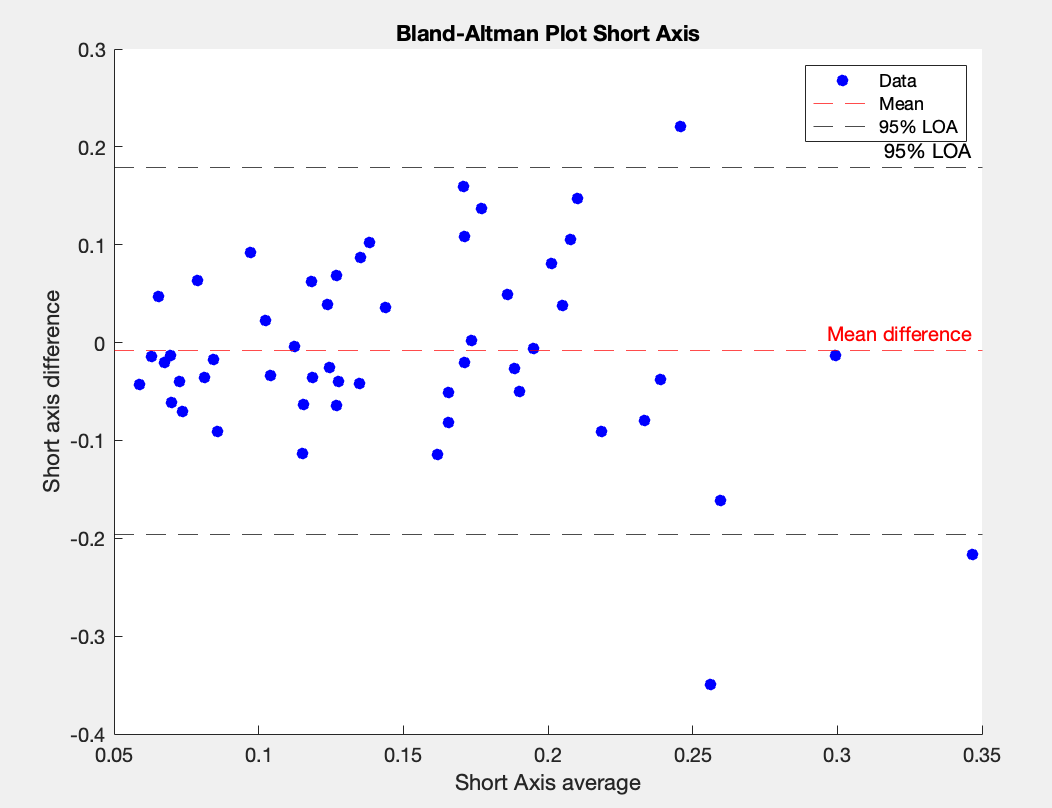

Supplement: Supplemental Information 5 [file peerj-14-20806-s005.png]

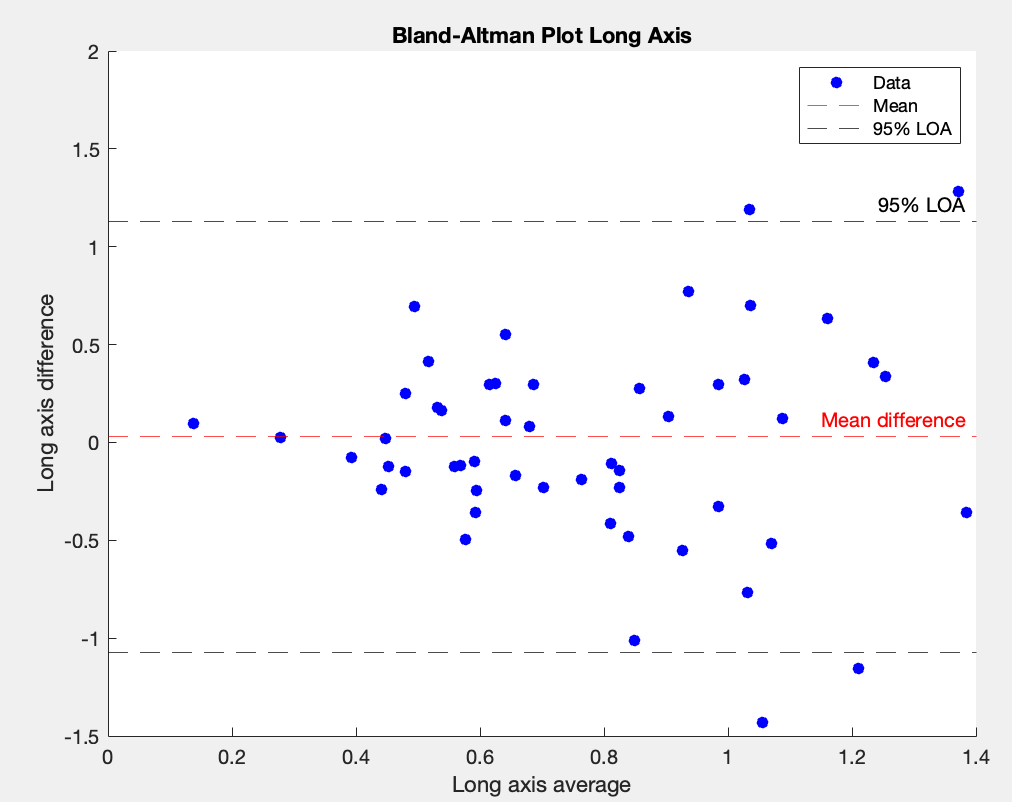

Supplement: Supplemental Information 6 [file peerj-14-20806-s006.png]
